# Parents’ Perspectives and Societal Acceptance of Implementation of Newborn Screening for SCID in the Netherlands

**DOI:** 10.1007/s10875-020-00886-4

**Published:** 2020-10-18

**Authors:** Maartje Blom, Robbert G. M. Bredius, Marleen E. Jansen, Gert Weijman, Evelien A. Kemper, Clementien L. Vermont, Iris H. I. M. Hollink, Willem A. Dik, Joris M. van Montfrans, Mariëlle E. van Gijn, Stefanie S. Henriet, Koen J. van Aerde, Wouter Koole, Arjan C. Lankester, Eugènie H. B. M. Dekkers, Peter C. J. I. Schielen, Martine C. de Vries, Lidewij Henneman, Mirjam van der Burg, on behalf of the SONNET-Study Group

**Affiliations:** 1 Department of Pediatrics, Laboratory for Pediatric Immunology, Willem-Alexander Children’s Hospital, Leiden University Medical Center, Albinusdreef 2, 2333 ZA Leiden, The Netherlands; 2 Centre for Health Protection, National Institute for Public Health and the Environment (RIVM), Bilthoven, The Netherlands; 3 Department of Pediatrics, Willem-Alexander Children’s Hospital, Leiden University Medical Center, Leiden, The Netherlands; 4 Department of Vaccine Supply and Prevention Programmes, National Institute for Public Health and the Environment (RIVM), Bilthoven, The Netherlands; 5 Department of Clinical Chemistry, IJsselland Hospital, Capelle aan den IJssel, The Netherlands; 6 Department of Pediatric Immunology and Infectious Diseases, Sophia Children’s Hospital, Erasmus MC, University Medical Center, Rotterdam, The Netherlands; 7 Department of Clinical Genetics, Erasmus MC, University Medical Center, Rotterdam, The Netherlands; 8 Department of Immunology, Laboratory Medical Immunology, Erasmus MC, University Medical Center, Rotterdam, The Netherlands; 9 Department of Internal Medicine, Section Clinical Immunology, Erasmus MC, University Medical Center, Rotterdam, The Netherlands; 10 Department of Pediatric Immunology and Infectious Diseases, Wilhelmina Children’s Hospital, University Medical Center Utrecht, Utrecht, The Netherlands; 11 Department of Genetics, University Medical Centre Groningen, Groningen, The Netherlands; 12 Department of Pediatric Immunology and Infectious Diseases, Amalia Children’s Hospital, Radboud University Medical Center, Nijmegen, The Netherlands; 13 Department of Human Genetics, Radboud University Medical Center, Nijmegen, The Netherlands; 14 Centre for Population Screening, National Institute for Public Health and the Environment (RIVM), Bilthoven, The Netherlands; 15 Department of Medical Ethics and Health Law, Leiden University Medical Center, Leiden, The Netherlands; 16 Department of Clinical Genetics and Amsterdam Reproduction & Development Research Institute, Amsterdam UMC, Vrije Universiteit Amsterdam, Amsterdam, The Netherlands

**Keywords:** Severe combined immunodeficiency, newborn blood screening, parental perspective, interviews, questionnaire study

## Abstract

**Purpose:**

While neonatal bloodspot screening (NBS) for severe combined immunodeficiency (SCID) has been introduced more than a decade ago, implementation in NBS programs remains challenging in many countries. Even if high-quality test methods and follow-up care are available, public uptake and parental acceptance are not guaranteed. The aim of this study was to describe the parental perspective on NBS for SCID in the context of an implementation pilot. Psychosocial aspects have never been studied before for NBS for SCID and are important for societal acceptance, a major criterion when introducing new disorders in NBS programs.

**Methods:**

To evaluate the perspective of parents, interviews were conducted with parents of newborns with abnormal SCID screening results (*N* = 17). In addition, questionnaires about NBS for SCID were sent to 2000 parents of healthy newborns who either participated or declined participation in the SONNET-study that screened 140,593 newborns for SCID.

**Results:**

Support for NBS for SCID was expressed by the majority of parents in questionnaires from both a public health perspective and a personal perspective. Parents emphasized the emotional impact of an abnormal screening result in interviews. (Long-term) stress and anxiety can be experienced during and after referral indicating the importance of uniform follow-up protocols and adequate information provision.

**Conclusion:**

The perspective of parents has led to several recommendations for NBS programs that are considering screening for SCID or other disorders. A close partnership of NBS programs’ stakeholders, immunologists, geneticists, and pediatricians-immunologists in different countries is required for moving towards universal SCID screening for all infants.

**Electronic supplementary material:**

The online version of this article (10.1007/s10875-020-00886-4) contains supplementary material, which is available to authorized users.

## Introduction

In the past decade, neonatal bloodspot screening (NBS) for severe combined immunodeficiency (SCID) has been introduced in several screening programs worldwide [1–5]. After addition to the Recommended Uniform Screening Panel (RUSP) in the USA, all states introduced SCID screening progressively, realizing nationwide screening for SCID in 2018 [6]. Even though the screening technique for SCID has been available for over a decade, implementation into screening programs is accompanied by many challenges due to the complexity of NBS programs. NBS encompasses more than a laboratory test and implementation includes adjustments in education, finances, logistics, politics, and culture [7–9]. Even if a high-quality test method is available, public uptake and parental acceptance of the test method are not guaranteed.

SCID is one of the most severe inherited disorders of the immune system characterized by severe T cell lymphopenia that is variably associated with an abnormal development of B- and/or natural killer (NK) cells [10]. Patients with SCID are usually born asymptomatic but develop life-threatening infections in the first months of life. Prompt clinical intervention with hematopoietic stem cell transplantation (HSCT) or gene therapy is required to prevent a fatal outcome for these patients [11]. Previous studies showed that early detection and treatment in the pre-symptomatic phase lead to higher survival rates [12–14]. NBS for SCID is based on the measurement of T cell receptor excision circles (TRECs) via (semi-)quantitative PCR. TRECs are circular DNA fragments formed during the T cell receptor gene rearrangement in the thymus serving as a marker for thymic output [15]. Low TREC levels indicate reduced numbers of recently formed T lymphocytes [16, 17]. To distinguish SCID from other T cell lymphopenias, follow-up diagnostics by flow cytometric immunophenotyping and genetic analysis are indicated [18].

Similar to other countries [19–23], the Netherlands started a prospective implementation pilot study (SONNET-study) in April 2018, focusing on parental perspective, cost-effectiveness, and practical implications for screening, diagnostics, and clinical follow-up. As parents are important stakeholders in NBS, their support is paramount. NBS pilot studies provide an invaluable opportunity to assess parental views on the potential benefits and harms of screening for newborns and their families [24]. In many cases, experts will assume that patients and families will automatically welcome perceived advances in the field. However, this is not necessarily the case and it is important to gauge family perceptions of these advantages. Therefore, we investigated the societal and psychosocial aspects through the eyes of parents of healthy newborns and parents who received an abnormal SCID screening result for their newborn. Our findings have led to important recommendations that can be valuable to other countries that consider implementation of SCID screening in their NBS program.

## Methods

For the SONNET-study, all parents of newborns born in three of the twelve provinces of the Netherlands (Utrecht, Gelderland and Zuid-Holland) were asked to participate in a research project on NBS for SCID (opt-out consent). All dried blood spots (DBS) included (*N* = 140,593) were collected as part of the Dutch routine NBS program from April 2018 to February 2020 (Figure S1). Demographic and clinical variables were collected from the national Praeventis NBS database (RIVM, Bilthoven, the Netherlands). The SONNET-study was approved by the Medical Ethics Committee of the Erasmus MC, University Medical Center, Rotterdam (MEC-2017-1146). TREC analysis was performed according to the SPOT-it™ kit instructions for use (ImmunoIVD, Stockholm, Sweden) according to a preset screening algorithm (Figure S2). From April 2018 to October 2018, a TREC cutoff value of ≤ 6 copies/3.2 mm punch was used. After 6 months of screening, the cutoff value was increased to ≤ 10 copies/3.2 mm punch from November 2018 to February 2020. A uniform diagnostic follow-up protocol and gene panel after abnormal TREC results was established (Figure S3; Table S1). Interviews were conducted with parents after an abnormal SCID screening result (*N* = 17). Items in the interview were evaluated either by categorical or non-categorical variables, the latter through open questions that were independently keyword-coded by two researchers to enhance the reliability of the results. The perspective of parents of healthy newborns on NBS for SCID who either participated (*N* = 1600) or declined participation (*N* = 400) in the SONNET-study was evaluated with a questionnaire that was specifically developed for this study by a multidisciplinary team of experts on NBS, medical ethics, and survey studies. The questionnaire was based on existing questionnaires previously used for investigating parents’ perspectives on NBS, e.g., for Pompe disease [25]. For qualitative validation and to address educational and language barriers, a small test phase was conducted to check for concept and wording of questions. The final concept was peer-reviewed before sending out. Construct validation questions were not included as it was not the goal to create a quantitative validated questionnaire about NBS for SCID. Practical barriers were addressed by offering parents the opportunity to send back a printed questionnaire or to fill in the questionnaire online by following a link or scanning a QR code. Multiple multivariate logistic regression analyses were performed to determine whether variables such as age, ethnicity, and educational level induced bias. For further details, see Methods in Supplemental data.

## Results

### TREC Screening and Referrals

**Fig. 1:**
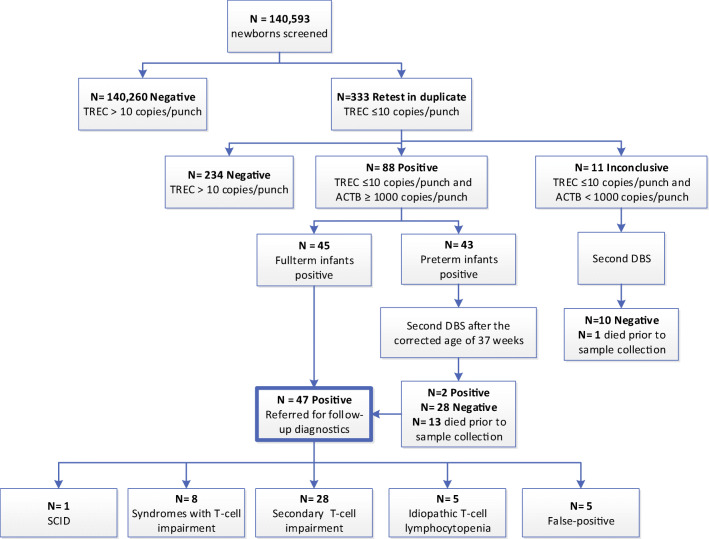
Number of referrals and retests based on TREC analysis. A total of 140,593 newborns were included for initial TREC analysis. NBS cards with TREC ≤ 10 copies/3.2 mm punch required repeated analysis in duplicate. Preterm: gestational age < 37 weeks and birth weight ≤ 2500 gram. Abnormal screening results with β-actin (ACTB) levels less than 1000 copies/3.2 mm punch were considered inconclusive and required repeated sampling (second DBS)

In total, 141,343 newborns participated in routine NBS in the pilot region. A total of 750 parents of newborns declined participation in the SONNET-study (participation rate 99.5%). Median TREC level in the study population was 97 copies/3.2 mm punch (IQR 66-141; Table S2). Receiving a blood transfusion less than 24 h prior to sample collection or early sample collection (< 72 h after birth) resulted in lower TREC levels (*P* < 0.05; Table S2). A total of 333 of the 140,593 newborns had TREC levels below the preset cutoff value after initial analysis (retest rate 0.24%; Fig. 1). In total, 47 full-term newborns with low TREC levels were referred for additional diagnostics (referral rate 0.03%; Fig. 1).

One SCID patient was identified with absent TRECs (0 copies/3.2 mm punch) and absent T cells. Genetic analysis revealed a pathogenic variant in the *IL2RG* gene (NM_000206.2(*IL2RG*):c.298C>T, p.(Gln100*)). The patient remained asymptomatic, underwent HSCT, and is currently in good clinical condition. In the other 46 newborns referred for further evaluation, five newborns had normal flow cytometric results with no known underlying cause for the low TREC levels (false-positive cases; Fig. 1). Of the 41 newborns with non-SCID T cell lymphocytopenia (TCL), eight infants had a congenital syndrome associated with T cell impairment, while five infants were reported to have idiopathic T cell lymphocytopenia with an unknown underlying cause (Table S3). In 28 cases, T cell lymphopenia could be attributed to other medical conditions without an intrinsic defect in the production of T cells (secondary T cell impairment; Table S3).

### Parents’ Experiences After an Abnormal SCID Screening Result

The parents of 23 newborns referred with an abnormal SCID screening result were approached for an interview, and 17/23 parents agreed (Table S4). Parents of eight newborns remembered receiving information about NBS for SCID prior to the heel prick and knowingly participated in the SCID pilot study. Nine parents did not remember receiving information, and one mother even questioned whether she would have participated in the SCID pilot study if she would have been formally asked.

Fifteen newborns were referred via the general practitioner (GP) to an academic medical center, while two newborns were already in the hospital at the time of referral. Referral via the GP is the standard procedure in the Dutch NBS program (Figure S1). Parents of twelve newborns experienced the referral procedure as negative, stating that they either received too little or incorrect information via the GP. In addition, parents experienced the initial counseling by the GP as unpleasant, for example, rushed via telephone contact instead of in person. Parents would have preferred to be contacted by a pediatric immunologist directly so they could receive correct and clear information from the start with the opportunity to ask questions. One couple appreciated being called by a familiar and trusted person as their GP, whereas two mothers who received the news via telephone stated that a personal visit from the GP would be excessive.

The majority of parents (15/17) were very satisfied with the rapid availability of the diagnostic results and the follow-up care provided by the pediatric immunologist. All parents stated to have experienced significant anxiety and emotional insecurity up to the visit in the hospital; however, their trust in the NBS program had not been changed by this experience.

Parental perception of the vulnerability of their newborn after definitive diagnosis was determined with the Vulnerable Baby Score (VBS) (*N* = 13). The mean VBS was 28.7 (SD4.8) compared with 23.1 (SD3.1) found in healthy control newborns [26] (Table 1). The mean total score of the parental stress questionnaire (OBVL) of these parents was 60.5 (SD 8.3) which is just above the norm for parents of children age category 0–3 years (Table 2). Parents experienced mild problems in the subcategory “restrictions to one’s own freedom and frustration in attempts to maintain one’s own identity” (T-score of 65.1) (Table 2).

**Table 1:** Vulnerable Baby Score (VBS) by parents of newborns with abnormal SCID screening results (*N* = 13)

	Number	Baby age when questionnaire completed in weeks Mean (range)	Mean VBS	SD
Healthy newborns	39*	13.4 (11.2–17.3)	23.1	3.1
Jaundice	19*	10.6 (9.6–14.1)	25.1	4.2
Medically fragile	17*	11.4 (9.5–15.0)	27.4	4.6
Newborns with abnormal SCID screening results (total)	13	21.2 (8.5–41.7)	28.7	4.8
T cell impairment syndromes	3		31.0	
Secondary T cell impairment	3		28.3	
Idiopathic lymphocytopenia	4		30.7	
False positive	3		25.7	

*Data of medically fragile, jaundice, and healthy control groups adopted from Kerruish et al. [26]

**Table 2:** Parental stress scores (OBVL) by parents of newborns with abnormal SCID screening results (*N* = 13)

	Number	Parent-child relationship problems	Parenting problems	Depressive mood	Parental role restriction	Physical Health problems	Total score
T cell impairment syndromes	3	62.7	63.7	63.0	64.0	68.3	68.7
Secondary T cell impairment	3	54.3	49.3	51.3	55.3	57.0	50.7
Idiopathic lymphocytopenia	4	57.0	58.8	60.8	71.2	65.8	63.5
False positive	3	54.3	50.3	58.7	67.3	56.7	58.0
Total	13	57.1	55.8	58.6	65.1	62.2	60.5

T-scores are the transformed raw data scores. Mild problems are implied with T-scores above 65 for the subcategories and a T-score above 60 for the total score

### Parental Perspective on NBS for SCID and Scientific Research on NBS

In total, 391 of 2000 parents of healthy newborns returned the questionnaire (response rate 19.6%). Of these parents, 84.9% (332/391) participated in the SONNET-study. Sixteen (4.1%) parents declined participation, and 33 (8.4%) parents could not remember whether they participated or not. The respondents’ characteristics are shown in Table S5 and Table S6. The mean age of respondents was 32.8 years (range 20–52 years). Most respondents were female (85.8%). Compared with the reference population (Table S5), respondents were higher educated and more likely to have a Dutch background.

Respondents in the questionnaire study were orally informed about NBS for SCID by the midwife/gynecologist (*N* = 107; 28.1%) and/or the screener (*N* = 181; 47.5%) (Table 3). Information provision by the midwife/gynecologist was rated best (evaluation score of 7.5). The majority of parents did not recollect to have received or did not read the information leaflet (*N* = 272; 72%). Parents who did receive the information leaflet were positive (evaluation score of 7.6). These parents indicated that the leaflet was clear (*N* = 98; 87.5%) and easy to read (*N* = 90; 80.3%) and that information was sufficient and understandable (Figure S4).

**Table 3:** Different information sources in the SCID pilot study and the evaluation scores of the received information by parents (*N* = 391)

Did you receive:	Yes, *N* (%)	No, *N* (%)	I do not remember/I do not know, *N* (%)	Evaluation score 1–10 (SD, *N*)			*P* value*
				Total	Participated in SCID pilot study	Declined participation in the SCID pilot study	
Oral information via the midwife/gynecologist	107 (28.1)	195 (51.2)	79 (20.7)	7.5 (SD 1.3, *N* = 128)	7.4 (SD 1.3; *N* = 118)	7.1 (SD 1.2; *N* = 6)	*P* = 0.537
Oral information via the screener	181 (47.5)	114 (29.9)	86 (22.6)	7.1 (SD 1.5, *N* = 196)	7.2 (SD 1.4; *N* = 175)	5.7 (SD 1.4; *N* = 12)	*P* = 0.001
Information leaflet				7.6 (SD 1.3, *N* = 112)	7.6 (SD 1.3; *N* = 110)	6.7 (SD 0.76; *N* = 7)	*P* = 0.019
From midwife/gynecologist	64 (18.2)	212 (60.4)	75 (21.4)				
From screener	59 (17.7)	202 (60.5)	73 (21.9)				
At city hall	22 (6.8)	230 (71.4)	70 (21.7)				
Visited the study website www.sonnetstudie.nl	13 (3.4)	368 (96.4)	1 (0.3)	7.8 (SD 1.6, *N* = 19)			

Missing values were excluded from the percentages. Evaluation scores were not individually calculated for parents who could not remember whether they participated in the SCID pilot study (*N* = 33)

*Mann-Whitney *U* test

Parents who declined participation in the SONNET-study were less positive about the provided information compared with parents who participated (Table 3). Participants were more likely to answer one of the knowledge questions correctly compared with parents who declined participation (*P* = 0.03) (Table S7).

Support for NBS for SCID was expressed by the majority of parents from a public health perspective “I think it is important that SCID is included in the newborn screening program” (rating mean 4.3) and a personal perspective “SCID is a severe disorder and I want this disorder to be detected as early as possible for my child” (rating mean 4.2; Table S8). Parents who declined participation in SCID screening had a more negative attitude towards scientific research in general (rating mean 3.5 versus 4.7 *P* < 0.01) and believed it to be of less importance that SCID is included in the NBS program (rating mean 2.9 versus 4.3 *P* < 0.01) (Table 4).

**Table 4:** Difference in attitude of parents who participated or declined participation in SCID screening (*N* = 348)

Questionnaire statement	Participated (*N* = 332)		Declined (N = 16)		*P* value*
	Rating mean (SD)^a^	% that agreed	Rating mean (SD)^a^	% that agreed	
“Scientific research”–related statements					
Scientific research is required to prevent diseases	4.7 (0.64)	98.8	3.5 (1.41)	75	*P* < 0.01
Scientific research is required to improve treatment of diseases	4.7 (0.66)	98.2	3.9 (1.09)	87.5	*P* < 0.01
I do not want to participate in scientific research	1.5 (0.79)	10.7	3.2 (1.11)	75	*P* < 0.01
“NBS for SCID”–related statements					
SCID is a severe disorder and I want this disorder to be detected in my child as early as possible	4.4 (0.76)	98.2	2.9 (1.03)	62.5	*P* < 0.01
I think it is important that SCID is included in the newborn screening program	4.3 (0.74)	99.4	2.9 (0.93)	75	*P* < 0.01
The person who performed the heel prick advised me to participate in SCID screening	1.5 (0.81)	14.2	1.3 (0.88)	0	*P* = 0.542
My family/partner wanted the SCID test to be performed for my child	2.8 (1.24)	66.6	1.8 (1.13)	25	*P* < 0.01
I only want my child tested for SCID once the study has been completed and SCID has been included in the newborn screening program	2.1 (1.11)	26.5	2.6 (0.96)	56.2	*P* = 0.025
“Health of their child”–related statements					
I want as much information as possible about my child’s health	4.1 (0.90)	94.5	2.9 (1.36)	56.2	*P* < 0.01
I want to be reassured that my child does not have SCID	4.0 (1.00)	91.5	2.7 (1.45)	50	*P* < 0.01
I do not worry about the health of my child	3.4 (1.19)	76.1	3.9 (1.24)	82.2	*P* = 0.047
I think I have a high risk of getting a child with SCID	1.8 (0.88)	26.9	1.4 (0.73)	12.5	*P* = 0.068

*SD* standard deviation

*Mann-Whitney *U* test

^a^Five-point rating scale: 1 = fully disagree; 5 = fully agree. Parents who could not remember whether they declined or participated (*N* = 33) and missing values (*N* = 10) are excluded from the percentages

### Reasons to Participate or Decline Participation in NBS for SCID

Reasons to participate in NBS for SCID included the potential health benefit for their child (41.8%), to support scientific research (41.8%), the fact that no extra blood had to be drawn (12.5%), the disorder can be cured (8.1%), and to help other children (6.6%) (*N* = 340). Parents who declined participation (*N* = 16) stated that they declined because of insufficient/misconception of information, a low a priori risk of the disease, the test still being in a research phase, not being interested in knowing or due to privacy reasons. Parents who read the leaflet/received information about the pilot study were not more likely to participate, but parents with higher knowledge scores were marginally more likely to participate in NBS for SCID (*P* = 0.06; Table S9). Respondents with one child (first-time parents) were more likely to participate in NBS for SCID compared with parents with more children (*P* = 0.04; Table S9).

## Discussion

NBS for SCID based on TREC quantification has been implemented in several countries; thus, the effectiveness of TREC quantification for SCID detection has been demonstrated [27, 28]. However, the availability of a high-quality test method does not automatically guarantee acceptance from the perspective of stakeholders such as parents. Therefore, our study focused on societal context including public awareness and understanding by studying the perspectives of parents and evaluating the practical aspects for screening, diagnostic procedures, and clinical follow-up. Psychosocial aspects have never been reported before in NBS for SCID while they are important for societal acceptance, a major criterion when introducing new disorders in NBS programs.

Interviews with parents revealed that parents experienced anxiety and stress when receiving an abnormal screening result for SCID. Most parents were informed by their GP and felt their GP lacked important knowledge about SCID while experiencing telephone contact as impersonal and rushed. International studies show that healthcare providers acknowledge the difficulty of delivering abnormal screening results to parents [29, 30]. Some providers deliberately keep information during this first contact to a minimum trying to reduce parental anxiety [31]. Communication scripts developed together with parents could help a primary healthcare provider in this first contact [29]. In the interviews, parents suggested tandem telephone calls with both their GP and a pediatric immunologist to provide support and expert information at the time of the referral. Most parents commended their experience with the pediatric immunologist and were relieved with the rapid availability of diagnostic results. The magnitude of parents’ distress while waiting for infants’ confirmatory test results should not be underestimated [30]. Similar to studies for NBS for cystic fibrosis (CF), all parents would still participate in NBS for SCID despite their experiences in the referral procedure [32–34]. Parents scored relatively high on the Vulnerable Baby Scale in comparison with parents of healthy newborns [26]. Even parents with a confirmed healthy newborn after follow-up (false-positive) perceived their newborn as more “vulnerable” implying some effect of the referral procedure with the associated feelings of anxiety [35, 36]. Parents additionally experienced some mild problems in their parental role. The interviews provide a more in-depth understanding of the impact of an abnormal SCID screening result for parents and emphasize the importance of reducing false-positive referrals.

Our questionnaire study amongst parents of healthy newborns showed that parents have a positive attitude towards NBS for SCID. Most parents stated that they wanted SCID to be detected as early as possible for their child. While our respondent group was different from the Dutch reference population, their opinion might still reflect the attitude of the general Dutch population. Other studies have also shown public support for expanded NBS and a positive attitude towards NBS in general [37–39]. As these studies also used self-developed surveys, one could argue that there is a need for a general validated questionnaire that evaluates parental perspectives on implementation of new disorders in NBS programs. First-time parents with only one child were more likely to participate in NBS for SCID than parents with more children. These findings were also observed in our previous questionnaire study in which “new” parents were more likely to participate in hypothetical NBS for the untreatable disorder ataxia telangiectasia, a potential incidental finding for NBS for SCID [40, 41]. The key motivator for parents for participation in NBS for SCID was to benefit the health of their own child, but also supporting scientific research and the non-invasive character of NBS for SCID were reported arguments. These findings are in accordance with previous studies in which reasons for accepting newborn screening were investigated [42–44].

Some parents declined participation in NBS for SCID due to insufficient information and misconception of the pilot study, illustrating the importance of providing adequate information in NBS programs. Our findings confirm previous research indicating that NBS education does not always reach parents and there is a persistent lack of public knowledge about NBS [37, 45]. These studies also showed that healthcare providers are the preferred source of NBS information, advocating for incorporation of NBS education into prenatal care and for midwifes to counsel parents [37, 45]. Information provision and timing of information in NBS have been ongoing topics of discussion with little consensus between countries [46]. Other means such as digital apps or videos should be explored in the near future.

In summary, our pilot study shows that while the central idea of early detection of SCID to facilitate treatment is simple, successful implementation of NBS for SCID is a complex process with parental acceptance being of great importance when introducing new disorders in NBS programs. The findings of this study on parental perspectives have led to several recommendations for other NBS programs that are considering SCID screening or future implementation of other disorders (Table 5).

**Table 5:** Recommendations for other NBS programs that are considering SCID screening or future implementation of other disorders

• Clear information provision by the indicated health care provider both prior to the NBS program as well as during the referral procedure after an abnormal screening result is of utmost importance for parents.	
• Tandem telephone calls by primary health care providers and pediatricians(-immunologists) should be considered when delivering the news about abnormal screening results to parents.	
• Follow-up care after an abnormal screening result, independent of the outcome/diagnosis, should be provided as parents can experience (long-term) stress and anxiety after a referral	
• All possible adaptations to the NBS leading to more targeted screening for the core condition SCID and the reduction of the number of incidental findings and false-positive cases should be explored.	
• Uniform follow-up protocols are required for a prompt and consistent approach to a definitive diagnosis and can provide guidance for pediatrician-immunologists when dealing with the relatively high number of incidental findings accompanied by NBS for SCID.	
• Parents’ perspectives should be taken into account when introducing new disorders in NBS programs as societal acceptance is of utmost importance.	
• A close partnership of NBS programs, patient organizations, immunologists, geneticists and HSCT specialists in different countries could help to promote standardization of care and follow-up protocols.	
• Shared learning should be facilitated internationally to support effective implementation of SCID screening suited to the local context to move towards universal harmonized SCID screening for all infants.	

## Electronic Supplementary Material

ESM 1 (PDF 575 kb)
