# Supplementary material for: Parents’ Perspectives and Societal Acceptance of Implementation of Newborn Screening for SCID in the Netherlands
Source: J Clin Immunol. 2020 Oct 18;41(1):99–108. doi: 10.1007/s10875-020-00886-4 (PMC7846522; doi:10.1007/s10875-020-00886-4)
Supplement: Supplementary file 1 — (PDF 575 kb) [file 10875_2020_886_MOESM1_ESM.pdf]

Supplemental data

Methods

Dutch NBS program

The Dutch NBS program screens for 22 disorders in 2020, performing approximately 170,000 blood spot tests each year. Parents receive information about NBS during pregnancy from the midwife/gynecologist during the first consultation and at 36-42 weeks. After birth, parents will receive additional information when they register their newborn at city hall and from screeners who perform the heel prick (Figure S1). Blood spot collection is primarily performed at home as soon as possible after 72 hours or after 96 hours (if combined with neonatal hearing screening) and no later than 168 hours (Figure S1). At the national level, the NBS program is coordinated and monitored by the National Institute for Public Health and the Environment (RIVM) [1].

TREC analysis

TREC analysis was performed according to the SPOT-it^{TM} kit instructions for use (ImmunoIVD, Stockholm, Sweden) in two screening laboratories (RIVM, Bilthoven and IJsselland Hospital, Capelle aan den IJssel). β-actin served as a control marker. Single 3.2 mm discs (equal to 3.0 μL blood) were punched from heel prick cards into a Filter plate using a Wallac DBS puncher (1296-071, PerkinElmer, Turku, Finland). Samples were rinsed and transferred into an Elution plate for elution at 95°C. Next, eluted DNA was transferred into a qPCR plate and analyzed in a QuantStudio 5 qPCR system (ThermoFisher, Waltham, Massachusetts, USA). Copy numbers were calculated with the QuantStudio software 1.4.2 (ThermoFisher).

Cut-off value and screening algorithm

From April 2018 to October 2018, newborns with TREC ≤ 6 copies/3.2 mm punch were referred for clinical follow-up, according to the kit instructions (ImmunoIVD). After six months of screening, the TREC cut-off value was increased to ≤ 10 copies/3.2 mm punch from November 2018 to February 2020 to ensure that no atypical SCID cases would be missed. For the complete screening algorithm, see Figure S2.

Diagnostic follow-up protocol

A uniform diagnostic follow-up protocol after abnormal TREC results was established (Figure S3). Clinical evaluation by a pediatrician-immunologist was realized within 72 hours after an abnormal TREC result. Immunophenotyping by flow cytometry included analysis of CD3+ T-cells, CD4+ and CD8+ T-cells, CD56/16 NK-cells and CD19 B-cells and T-cell subsets CD45RA/CD45RO (%) naive T-cells. Newborns with normal levels of T-cells and without underlying cause for low TREC levels were considered false positive and follow-up was not conducted. In the case of absent or low/abnormal T-cells, initial genetic analysis was performed by whole-exome sequencing and subsequent gene panel analysis of the known causative genes for SCID (Table S1). Newborns with absent T-cells would simultaneously start with the HSCT work-up and while awaiting HSCT, protective measures would be taken. In the case of low or abnormal T-cells, additional immunological diagnostics could be carried out.

Follow-up interviews with parents after an abnormal TREC result

Interviews were conducted with parents after an abnormal SCID screening result to explore what parents experienced during the referral procedure. The semi-structured interview-guide included six topics: 1) views on information about the SONNET-study and information provision during referral, 2) reasons to participate in the SONNET-study, 3) experiences with referral procedure and the follow-up care after an abnormal screening result, 4) trust in the NBS program and 5) current clinical condition of the newborn and 6) psychological wellbeing of the parents. In total, 23 parents were approached for an interview. Parents of newborns who had deceased and parents of newborns who were in the hospital at the time of the referral were not contacted (N=24). After the interview, parents were asked to complete two questionnaires, the Vulnerable Baby Scale [2] and parental stress OBVL (Opvoedingsbelasting Vragenlijst) questionnaire to asses parental perception of their newborn and themselves. Interviews were either conducted in person or by telephone. The interviews were recorded and transcribed verbatim. Transcripts were coded independently by two researchers using the software MAXQDA 2018 0-5 (VERBI GmbH, Berlin, Germany).

Questionnaire study on parents' perspective on NBS for SCID

To evaluate the perspective of parents on NBS for SCID, a questionnaire was sent to 2000 parents of healthy newborns born in the pilot provinces; N = 400 parents who declined participation in the SONNET-study and N = 1600 parents who participated. Parents were able to send back a printed questionnaire or to fill in the questionnaire online by following a link or scanning a QR-code. Data were

collected and analyzed anonymously. Due to privacy reasons, it was not allowed to send reminders. The questionnaire was subdivided into four different sections: 1) opinion on the provided information in the SONNET-study, 2) knowledge about SCID, 3) opinion on NBS for SCID, and 4) demographic information (gender, age, ethnicity, educational level, number of children).

Data analyses

Descriptive statistics were used to summarize the distribution of TREC and β-actin levels in the Dutch newborn pilot population, and the characteristics of the respondents of the questionnaire study. Mann-Whitney U and Kruskal-Wallis tests were used for group comparison. The sociodemographic characteristics of the questionnaire respondents were compared to the Dutch reference population with one sample-*t*-test for age, chi-square test for trend for number of children and Pearson's chi-square test for other variables. Ordinal variables from scaled items were reported as means. Odds ratios and multivariate logistic regression analyses were used to determine associations between sociodemographic variables and outcome variables such as participation in the SONNET study. By assigning 1 point for each correctly answered knowledge question, an overall knowledge score was created (0-4). *P*-values < 0.05 were considered statistically significant. Statistical analysis was carried out with SPSS version 25.0 for Windows (SPSS, Inc., Chicago, IL, USA).

References

1. Blom, M., et al., *Introducing Newborn Screening for Severe Combined Immunodeficiency (SCID) in the Dutch Neonatal Screening Program.* International Journal of Neonatal Screening, 2018. **4**(4): p. 40.
2. Kerruish, N.J., et al., *Vulnerable Baby Scale: development and piloting of a questionnaire to measure maternal perceptions of their baby's vulnerability.* J Paediatr Child Health, 2005. **41**(8): p. 419-23.
3. Statistics Netherlands. *Birth; key figures*. 2017 [cited 2019 15 July]; Available from: https://opendata.cbs.nl/statline/#/CBS/en/dataset/37422eng/table?ts=1564486871296.
4. Statistics Netherlands. *Population; key figures*. 2018 [cited 2019 15 July]; Available from: https://opendata.cbs.nl/statline/#/CBS/en/dataset/37296eng/table?ts=1564487099871.
5. Statistics Netherlands. *Households; size, composition, position in the household, 1 January*. 2018 [cited 2019 15 July]; Available from: https://opendata.cbs.nl/statline/#/CBS/en/dataset/82905ENG/table?ts=1564487413252.
6. Statistics Netherlands. *Labour Force; level of education by personal characteristics*. 2019 [cited 2019 15 July]; Available from: https://opendata.cbs.nl/statline/#/CBS/en/dataset/71822eng/table?fromstatweb.
7. RIVM. 2017 2017; Available from: https://draaiboekhielprikscreening.rivm.nl/documenten/stroomschema-uitvoering-hielprikscreening.
8. Dorsey, M.J., et al., *Treatment of infants identified as having severe combined immunodeficiency by means of newborn screening.* J Allergy Clin Immunol, 2017. **139**(3): p. 733-742.

Tables

Table S1. SCID gene panel used in the follow-up after an abnormal TREC-result

Genes included in SCID gene panel	OMIM gene
ADA	608958
AK2	103020
B2M	109700
CD247	186780
CD3D	186790
CD3E	186830
CD3G	186740
CD8A	186910
CIITA	600005
CORO1A	605000
DCLRE1C	605988
DOCK2	603122
DOCK8	611432
FOXN1 (added per 01-01-2020)	600838
IL2RG	308380
IL7R	146661
JAK3	600173
LAT	602354
LCK	153390
LIG4	601837
NHEJ1	611290
PNP	164050
PRKDC	600899
PTPRC	151460
RAC2	602049
RAG1	179615
RAG2	179616
RFX5	601863
RFXANK	603200
RFXAP	601861
RMRP	157660
STK4	604965
TAP1	170260
TAP2	170261
TAPBP	601962
TTC7A	609332
ZAP70	176947

Table S2. Demographic variables and SCID screening parameters in the Dutch newborn pilot population

**Sample size**	**N = 140,593**	**TRECs in copies/3.2 mm punch**<br><br>Median (IQR)	**β-actin in copies/3.2 mm punch**<br><br>Median (IQR)
All newborns		97 (66-141)	3,564 (2,455-5,079)
Sex<br><br>Male<br>Female	 <br>51.4% (N = 72,209)<br>48.6% (N = 68,375)<br><br>Missing 10	 <br>92 (62-133)<br>103 (70-148)<br><br>P < 0.01	 <br>3,494 (2,409-4,981)<br>3,641 (2,502-5,194)<br><br>P < 0.01
Age at sample collection (in hours), median (IQR)<br><br>Early collection <72 hours<br>Timely sample collection (72 to 168h)<br>Late collection >168 hours	102 (89-121)<br><br><br>0.9% (N = 1,181)<br>97.4% (N =136,865)<br>1.7% (N = 2,544)<br><br>Missing 3	 <br><br>86 (59-124)<br>97 (66-140)<br>123 (82-179)<br><br>P < 0.01	 <br><br>3,816 (2,633-5,454)<br>3,564 (2,455-5,077)<br>3,503 (2,378-5,048)<br><br>P < 0.01
Gestational age (in days), median (IQR)<br><br>Extremely preterm <32 weeks<br>Preterm (32-36 weeks)<br>Term ≥ 37 weeks	278 (271-285)<br><br><br>1.1% (N = 1,483)<br>5.4% (N = 7,643)<br>93.5% (N = 131,399)<br><br>Missing 68	 <br><br>59 (34-95)<br>85 (56-123)<br>99 (67-142)<br><br>P < 0.01	 <br><br>3,750 (2,342-5,932)<br>3,374 (2,324-4,835)<br>3,576 (2,465-5,085)<br><br>P < 0.01
Birth weight (in grams), median (IQR)<br><br>Low birth weight <2500 gram<br>Normal birth weight ≥2500 gram	3450 (3,105-3780)<br><br><br>5.5% (N = 7,759)<br>94.5% (N = 132,808)<br><br>Missing 26	 <br><br>81 (52-121)<br>98 (67-142)<br><br>P < 0.01	 <br><br>3,460 (2,362-5,093)<br>3,571 (2,461-5,078)<br><br>P < 0.01
Blood transfusion <24 hours sample collection<br><br>Yes<br>No	 <br><br>0.01% (N = 32)<br>99.9% (N = 140,561)<br><br>Missing 0	 <br><br>44 (20-77)<br>97 (66-141)<br><br>P < 0.01	 <br><br>3,650 (2,466-5,518)<br>3,564 (2,455-5,079)<br><br>P = 0.596

Notes: TRECs - T-cell receptor excision circles, IQR – interquartile range. Mann-Whitney U test was used for gender, birth weight and <24 hour blood transfusion comparison with TREC/β-actin levels. Kruskal-Wallis test for was used for comparison between age at sample collection and gestational age with TREC/β-actin levels.

Table S3. Diagnoses of 47 infants with (non)-SCID T-cell lymphopenia and false-positive results identified via newborn screening

Classification		Number of newborns<br><br>(N = 47)	TRECs in copies/3.2 mm punch<br><br>Median (range)
**SCID**	X-linked SCID (*IL2RG*)	1	0
**T-cell impairment syndromes**	22q11.2 deletion syndrome (DiGeorge)	4	1.5 (0-8)
	Trisomy 21	2	4.5 (1-10)
	Noonan syndrome	1	0
	Heterozygous *FOXN1* variant	1	6
**Secondary T-cell impairment**	Multiple congenital anomalies[a]	7	8 (2-13)
	Congenital diaphragmatic hernia	3	7 (2-10_
	Cardiac anomalies	2	8.5 (6-15)
	Gastrointestinal anomalies	2	2 (0-8)
	Chylothorax and hydrops	1	0
	Sepsis/severe infections	6	5.5 (0-18)
	Maternal immunosuppressant use	3	5 (1-16)
	Other neonatal conditions[b]	4	6 (2-9)
**Idiopathic T-cell lymphocytopenia**		5	5 (2-26)
**False-positive**		5	5 (0-14)

a. Multiple congenital anomalies included newborns with nemaline rod myopathy (de novo variant *ACTA1*), holoprosencephaly/diaphragmatic hernia due to *GLI1* variant, MADD deficiency and others.

b. Other neonatal conditions included severe asphyxia, dysmaturity, high doses of dexamethasone and start of chemotherapeutics prior to sample collection.

Table S4. Characteristics of the interviewees after an abnormal TREC result (N = 17)

Respondent #	Diagnosis after follow-up	Interview setting	Interview with	Referral via
1	22q11.2 deletion syndrome/DiGeorge	Academic medical center	Mother	General practitioner
2	Lymphopenia due to severe sepsis	Telephone	Father	Hospital
3	Mother used immunosuppressant medication during pregnancy	Home	Mother	General practitioner
4	Mother used immunosuppressant medication during pregnancy	Academic medical center	Both parents	General practitioner
5	Idiopathic T-cell lymphocytopenia	Home	Mother	General practitioner
6	False positive	Home	Mother	General practitioner
7	Idiopathic T-cell lymphocytopenia	Home	Mother	General practitioner
8	Idiopathic T-cell lymphocytopenia	Home	Both parents	General practitioner
9	False positive	Telephone	Mother	General practitioner
10	Idiopathic T-cell lymphocytopenia	Telephone	Mother	General practitioner
11	Mother used immunosuppressant medication during pregnancy	Telephone	Mother	General practitioner
12	False positive	Telephone	Mother	General practitioner
13	False positive	Telephone	Mother	General practitioner
14	22q11.2 deletion syndrome/DiGeorge	Telephone	Mother	General practitioner
15	Noonan syndrome	Telephone	Mother	Hospital
16	Idiopathic T-cell lymphocytopenia	Telephone	Mother	General practitioner
17	False positive	Telephone	Mother	General practitioner

Table S5. Sociodemographic characteristics of the questionnaire respondents (N = 391)

Variables	Respondents questionnaire study	Reference group Dutch population	P-value
	N = 391	N (x1000)	
Age in years (range)		Dutch parents[a]	
Mean age mothers	31.8 (20-45)	31.4	P = 0.401
Mean age fathers	35.0 (26-52)	34.2	P = 0.075
Missing	20		
Gender, N (%)		Dutch population age 20-50 years[b]	P <0.01
Female	319 (85.8)	3,266 (49.7)	
Male	53 (14.2)	3,304 (50.3)	
Missing	19		
Background, N (%)		Dutch population age 20-50 years[c]	P <0.01
Dutch	312 (83.9)	4,675 (70.6)	
Other	60 (16.1)	1,932 (29.4)	
Missing	19		
Civil registry, N (%)		Dutch population age 20-50 years[d]	P <0.01
Living together/Married	362 (97.3)	2,024 (78.4)	
Single	10 (2.7)	572 (21.6)	
Missing	19		
Highest level of education, N (%)		Dutch population age 20-50 years[e]	P <0.01
Low	18 (4.9)	585 (30.9)	
Middle	101 (27.2)	1,643 (38.1)	
High	252 (67.9)	1,908 (29.4)	
Missing	20		
Number of children, N (%)		Dutch parents[f]	P = 0.17
1	181 (48.7)	71.9 (44.2)	
2	129 (34.7)	62.5 (38.5)	
≥3	62 (16.6)	28.1 (17.3)	
Missing	19		

Missing values were excluded from the percentages.

a. Reference population Dutch Parents [3]. One sample T-test.

b. Reference population Dutch population age 20-50 years [4]. χ2 test

c. Background was coded as ‘Dutch’ if both parents were born in the Netherlands. Reference population Dutch population age 20-50 years [4]. χ2 test

d. Reference population Dutch population households [5]. χ2 test

e. Low: primary education, lower vocational education, lower and middle general secondary education. Middle: middle vocational education, higher secondary education, and pre-university education. High: higher vocational education and university. Reference population Dutch population age 25-45 years [6]. χ2 test

f. Reference population Dutch parents [5]. χ2 test for trend.

Table S6. Participation in NBS, health status of the children and familial disorders of the questionnaire respondents (N = 391)

	Respondents questionnaire study<br><br>N (%)
Did all your children participate in the Dutch NBS program?	
Yes	364 (96.6)
No	5 (1.4)
Missing	22
What was the NBS result for your child(-ren)?	
Normal	364 (99.4)
Abnormal	1 (0.3)
I would rather not say	1 (0.3)
Missing	25
Are your children healthy? [a]	
Yes	362 (97.3)
No	8 (2.2)
I would rather not say	2 (0.5)
Missing	19
Do you have a family member with a hereditary disorder? [b]	
Yes	51 (13.7)
No	292 (78.7)
I do not know	26 (7.0)
I would rather not say	2 (0.6)
Missing	20

Missing values were excluded from the percentages.

a. Answers included a wide variety of hereditary disorders including Down Syndrome, trisomy 18, asthma, neurological and congenital anomalies.

b. Answers included a broad spectrum of disorders such as malignancies, metabolic diseases, diabetes mellitus, cardiovascular diseases, inflammatory bowel disease and autoimmune disorders.

Table S7. Knowledge questions about SCID and the percentage of parents answering them correctly based on participation in the SONNET study (N = 348)

Knowledge questions	Participated in the SONNET-study<br><br>N = 332	Declined participation in the SONNET-study<br><br>N = 16	
	% (N) that answered correctly:	% (N) that answered correctly:	P-value*
1. In the Netherlands, approximately 200 children are born with SCID each year.<br><br>*False*	75.4% (248)	75.0% (12)	0.568
2. Children with SCID get severe infections during the first months after birth.<br><br>*True*	93.3% (307)	93.8% (15)	0.711
3. The treatment for SCID is stem cell transplantation.<br><br>*True*	92.7% (305)	75.0% (12)	0.032
4. SCID can be cured if detected in an early phase.<br><br>*True*	90.6% (298)	81.3% (13)	0.201

Missing values (N = 10) and parents who could not remember whether they participated (N = 33) are excluded from the calculations. *Fisher exact test

Table S8. **Parental support of scientific research and NBS for SCID (N = 377)**

Questionnaire statement	Degree of support[a] (%)			Rating mean (SD)
	(Fully) disagree	Neutral	(Fully) agree	
Scientific research is required to prevent diseases	2.6	4.3	93.1	4.6 (0.75)
Scientific research is required to improve treatment of diseases	2.4	2.4	95.2	4.6 (0.72)
SCID is a severe disorder and I want this disorder to be detected in my child as early as possible	3.7	13.1	83.1	4.3 (0.90)
I think it is important that SCID is included in the newborn screening program	2.1	17.6	80.2	4.2 (0.82)
I want as much information as possible about my child's health	7.7	15.7	76.5	4.0 (0.96)
I want to be reassured that my child does not have SCID	9.9	20.3	69.9	3.9 (1.05)
I do not worry about the health of my child	23.0	22.0	54.9	3.4 (1.19)
My family/ partner wanted the SCID test to be performed for my child	36.0	39.8	24.2	2.7 (1.23)
I only want my child tested for SCID once the study has been completed and SCID has been included in the newborn screening program	69.6	14.8	15.7	2.2 (1.13)
I think I have a high risk of getting a child with SCID	71.7	27.4	0.8	1.8 (0.87)
I do not want to participate in scientific research	85.8	9.1	5.0	1.6 (0.90)
The person who performed the heel prick advised me to participate in SCID screening	85.1	13.0	1.9	1.5 (0.81)

SD = Standard deviation. [a] Five-point rating scale: 1 = fully disagree; 5 = fully agree converted into three-point scale. Missing values are excluded from the percentages.

Table S9. Variables and the likelihood of participation in pilot study: multivariate logistic regression analysis

	Participation in pilot study for NBS SCID (yes)		
Predictor variable	β	SE	P-value
Age	-0.031	0.059	0.593
Gender (female)	0.626	0.800	0.434
Ethnicity (Dutch)	0.797	1.087	0.464
Civil registry (Living together/married)	0.824	1.502	0.584
Educational level (high)	0.989	0.611	0.105
**Number of children**	**-0.593**	**0.294**	**0.044**
Having a child with a disorder (yes)	0.138	1.555	0.930
Having a family member with a hereditary disease (yes)	0.341	0.824	0.679
Received information via screener/midwife (yes)	-0.113	0.591	0.848
Read leaflet (yes)	-0.740	0.585	0.206
Knowledge score	0.625	0.335	0.062

Multivariate logistic regression analysis (N = 269 valid cases included for analysis) with standardized regression coefficients β and standard error (SE). In **bold** the significant predictor variable for participation in the SCID pilot study.

Figures

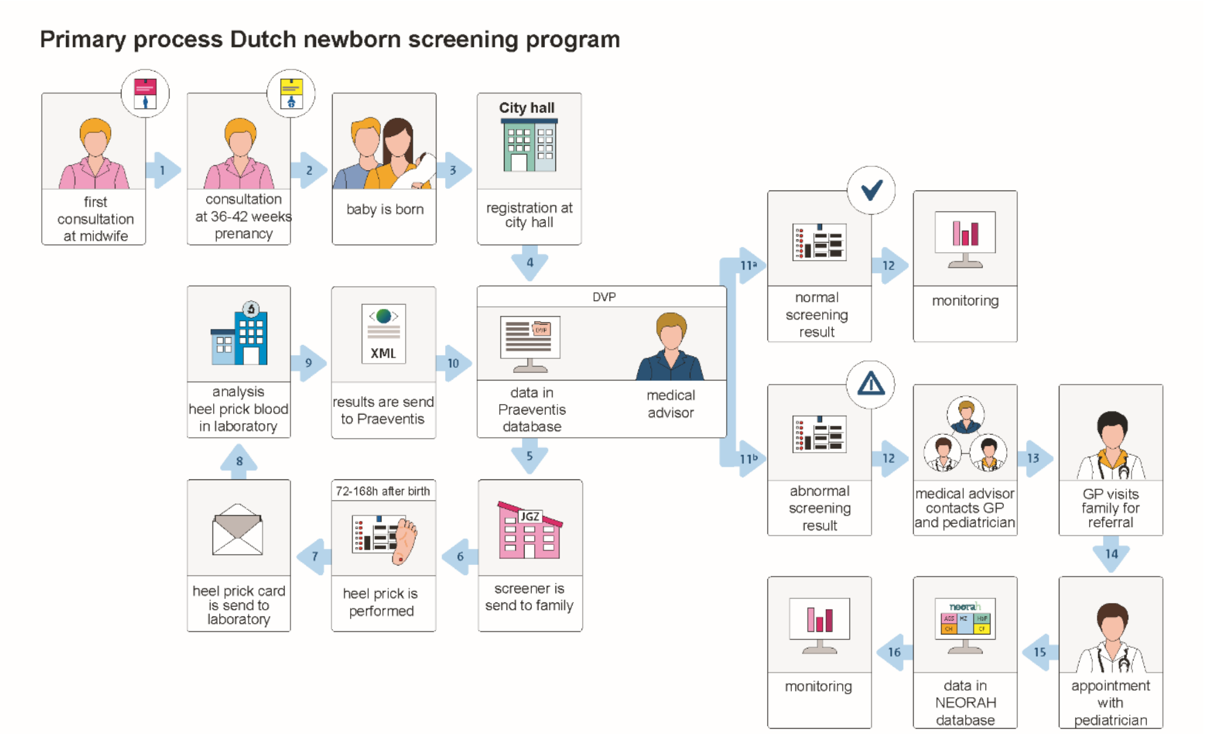

Figure S1. Primary process Dutch newborn screening program. Figure available via RIVM-website [7]. Expecting parents will receive information about the NBS program during the first and second consultation with the midwife (1-2). During registration at city hall, an information brochure will be handed out as well (3). The screening organizations (JGZ) will be informed about the registration of the newborn, after which screeners will visit the family to perform the heel prick (6). The heel prick card is send by post to one of the five screening laboratories (7). The heel prick cards are then analyzed and the results are registered in the national monitoring database Praeventis (8-9). Abnormal results are forwarded to the general practitioner (GP) and pediatrician by the medical advisor (12-13). Medical advisors coordinate logistics of the referral procedure. GPs will visit the family to inform them about the referral after which the family will visit the pediatrician for follow-up diagnostics (14).

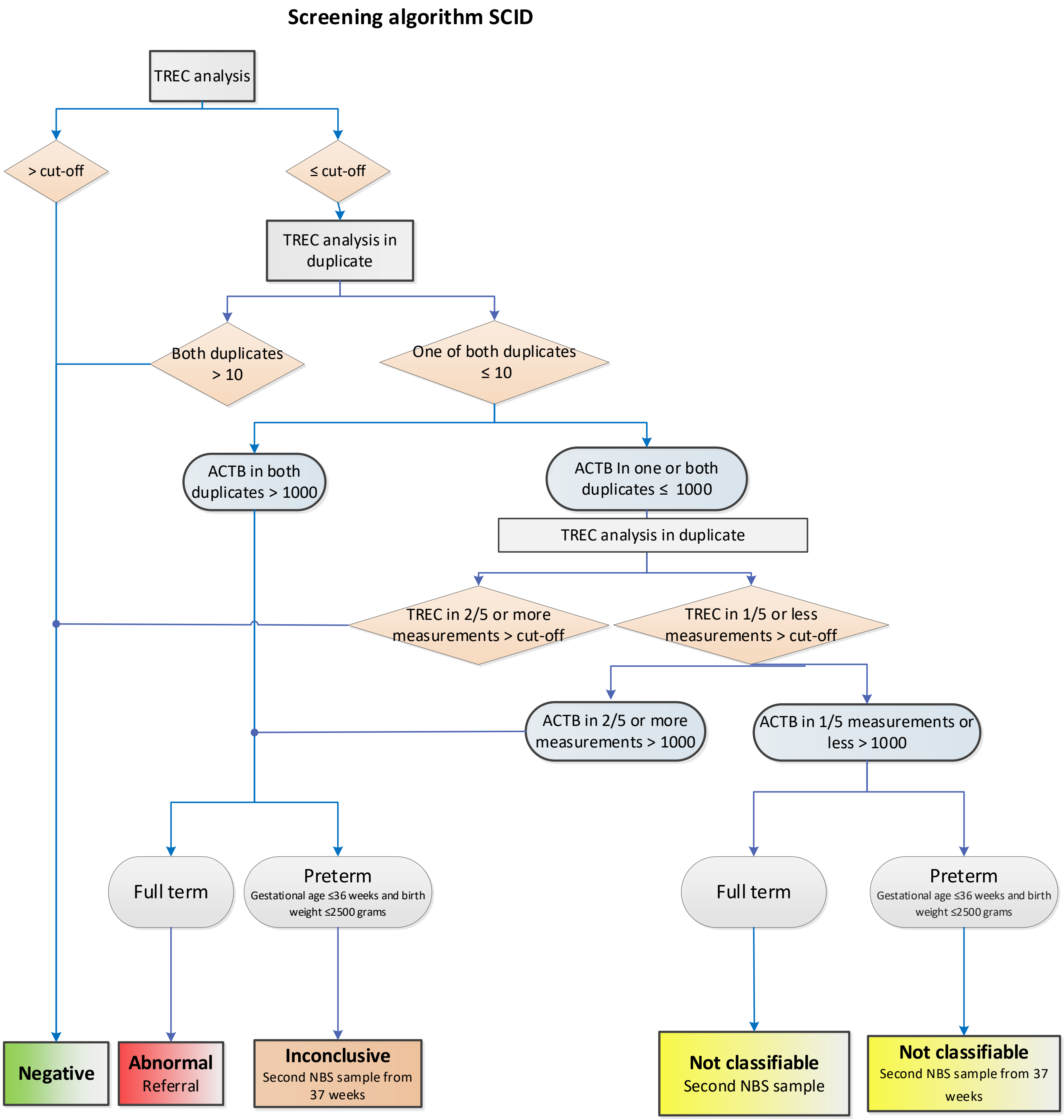

Figure S2. SCID screening algorithm. Samples with low TREC levels require repeated analysis in duplicate. Full term infants with low TREC levels were referred to an academic medical center for follow-up diagnostics. Preterm infants with abnormal results required a second specimen to be collected from the corrected age of 37 weeks.

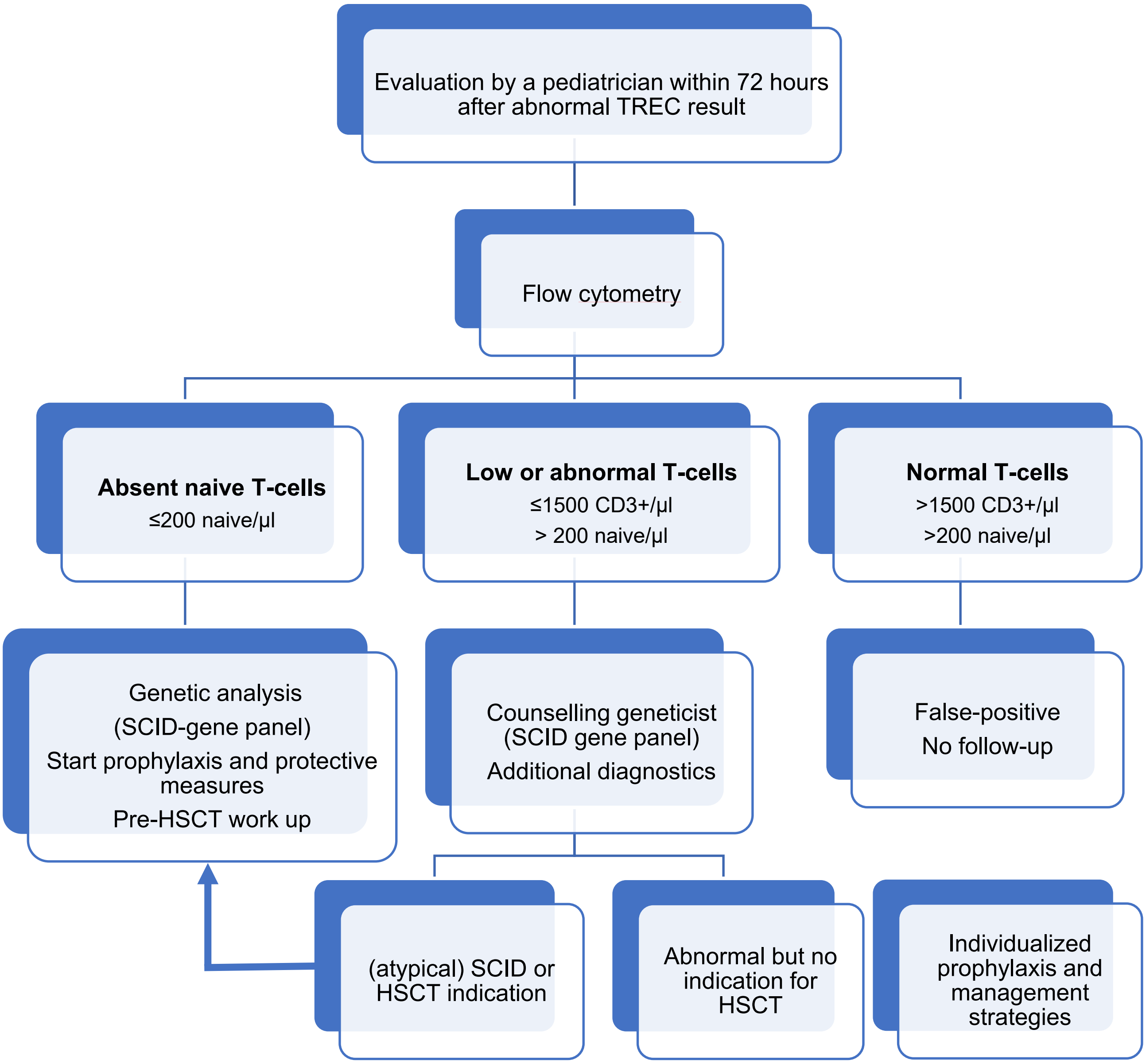

Figure S3. Uniform follow-up protocol used after an abnormal SCID screening result. Absent T-cells were defined as ≤200 naive CD4+ T-cells/µl blood. Low or abnormal T-cells were defined as ≤1500 CD3+ positive T-cells/µl blood >200 CD4+ naive T-cells/µl blood. Normal T-cells were defined as >1500 CD3+ positive T-cells/µl blood >200 CD4 naive T-cells/µl blood (in accordance with Dorsey *et al*. 2017 [8].

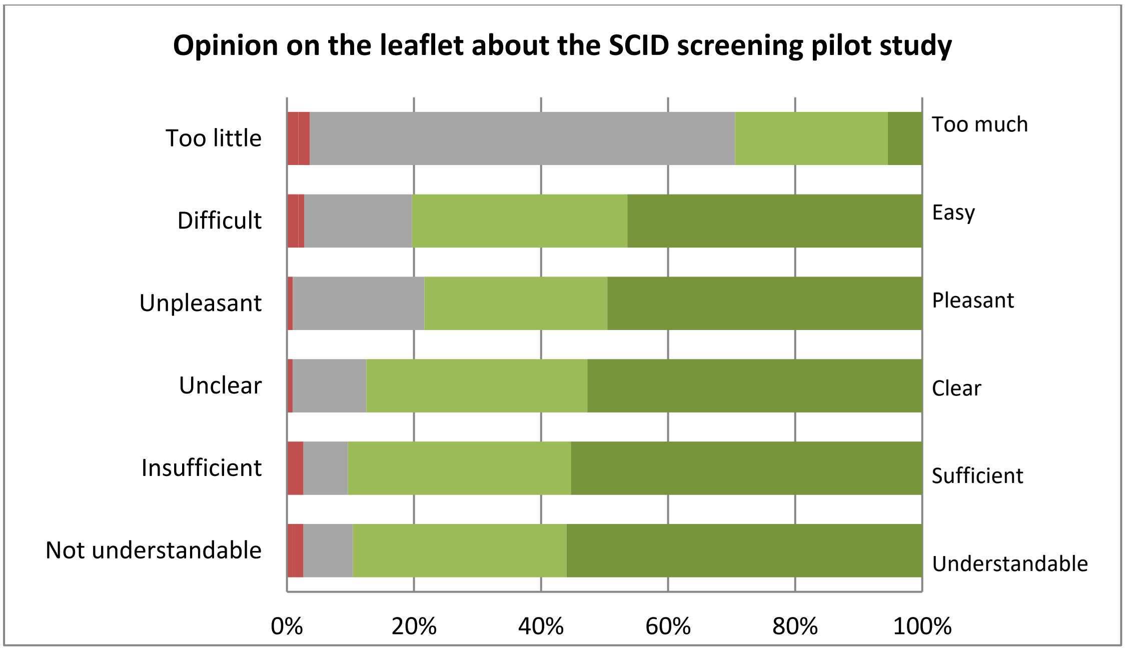

Figure S4. Opinion of parents on the leaflet about the SCID screening pilot study (N = 118). Only parents who answered that they read the leaflet were routed to this question**.** The red bars represent the percentage of parents who (totally) agreed with the words on the left side of the figure. The gray bars represent the percentage of participants with a neutral opinion towards the word pairs. The green bars represent the percentage of parents who (totally) agreed with the words on the right side of the figure.
